# Multiple analytical methods for determination of formoterol and glycopyrronium simultaneously in their novel combined metered dose inhaler

**DOI:** 10.1186/s13065-019-0592-9

**Published:** 2019-06-10

**Authors:** Yomna A. Salem, Mohammed E. A. Hammouda, Mohamed A. Abu El-Enin, Saadia M. El-Ashry

**Affiliations:** 1 Medicinal Chemistry Department, Faculty of Pharmacy, Mansoura University, Mansoura, 35516 Egypt; 2 Pharmaceutical Chemistry Department, Faculty of Pharmacy, Horus University-Egypt (HUE), New Damietta, Egypt

**Keywords:** Formoterol fumarate (FF), Glycopyrronium bromide (GLY), Ratio derivative spectrophotometry, Ion pair chromatography

## Abstract

One of the major causes of mortality all over the world is chronic obstructive pulmonary disease (COPD). Recently approved combined inhaler of formoterol fumarate (FF) and glycopyrronium bromide (GLY) has been used in very low concentrations (µg level/actuation) doses in COPD patients. The first spectrophotometric and advanced highly sensitive liquid chromatography has been achieved successfully throughout this study, permitting validated analysis of dual combined inhaler in raw material as well as pharmaceutical inhaled dosage form. Three sensitive analytical methods were carried out for the simultaneous assay of FF and GLY in their novel combined Metered dose inhaler (MDI). The first method depends on measuring the first derivative amplitudes at 208.27 nm for FF and at 213.27 nm and 239.86 nm for GLY, respectively. The second method depends on measurement of the first derivative of the ratio spectra at 214 or 229 nm for FF and 240 or 259 nm for GLY, respectively. For the spectrophotometric methods, the linearity ranges were 0.48–9.6 µg/mL for FF and 0.9–18 µg/mL for GLY. For the third method, valid ion-pairing chromatographic method was carried out applying C_18_ column and isocratic mobile phase of 60% v/v acetonitrile and 40% v/v deionized waster (pH 3.0) enclosing 0.025% sodium dodecyl sulfate, using UV detection adjusted to 210 nm and flow rate of 1.2 mL/min. For the ion-pairing chromatographic method, the linearity ranges were 0.048–4.8 µg/mL for FF and 0.09–9.0 µg/mL for GLY. The developed methods are reproducible, valid and offer efficient resolution between formoterol and glycopyrronium using spectrophotometric methods and highly sensitive and precise chromatographic method. The percent recoveries of the inhaled drugs in their MDI were good. The method was successfully established for the quantitative analysis of FF and GLY in their combined pharmaceutical inhaler capsules to validate the therapeutic efficiency of the combined drugs in quality control labs.

**Electronic supplementary material:**

The online version of this article (10.1186/s13065-019-0592-9) contains supplementary material, which is available to authorized users.

## Introduction

Chronic obstructive pulmonary disease (COPD) represents a serious health problem affecting millions of people and it is the third causing of death in the world after ischemic heart diseases and stroke. The recommended guidelines for COPD therapy including long acting bronchodilator or muscarinic antagonist [1].

**Fig. 1:**
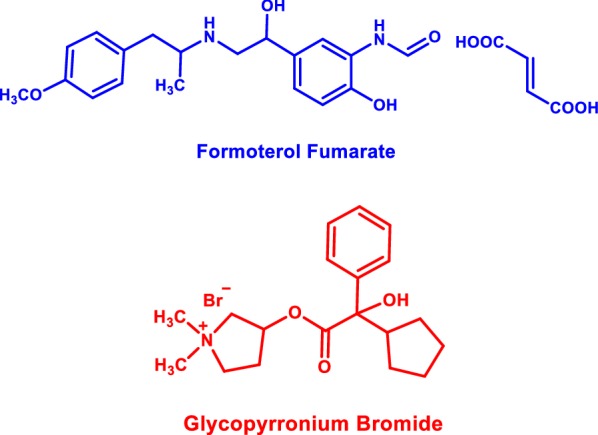
Structural formula of formoterol fumarate and glycopyrronium bromide

Formoterol fumarate(FF), *N*-[2-hydroxy-5-[(1*R*)-1-hydroxy-2-[[(2*R*)-1-(4-methoxyphenyl)propan-2-yl]amino]ethyl]phenyl]formamide, is an effective long-acting β_2_-adrenoceptor agonist [2] (Fig. 1). It is used for relaxation of smooth muscles in asthma and chronic obstructive pulmonary disease (COPD) [3]. Glycopyrronium bromide (GLY), 3-[(cyclopentylhydroxyphenylacetyl)oxy]-1,1-dimethyl-pyrrolidinium bromide [2], is a recently marketed long-acting muscarinic antagonist for maintenance treatment in COPD patients [4] (Fig. 1). Recently, the dual-bronchodilator mixture of FF and GLY is the recommended therapeutic choice for COPD patients due to its potent clinical efficacy and limited intake inhalation regimen [5].

Reviewing the literature survey, several methods were carried out for estimation of formoterol fumarate including spectrophotometry [6, 7], ultra performance liquid chromatography (UPLC) [8], gas chromatography mass spectrometric method (GC–MS) [9, 10], liquid chromatography [11–18], capillary electrophoresis [19–21]. Regarding, glycopyrronium bromide, few methods were described, including: UV [22, 23], conductometry [24], classical GC [25], HPLC [26–29], and advanced HPLC–MS methods [30, 31].

Formoterol fumarate and glycopyrronium bromide are co-formulated in MDI to relieve severe COPD patients [5]. It is challenging to resolve FF and GLY in the same chromatographic elution time as they have different lipophilic character that is difficult to separate in the same run. GLY is less lipophilic than FF as given by their log p values (log p values are − 1.2 and 1.91, respectively) [32].

Up till now, no spectrophotometric method has been described for the simultaneous assay of formoterol fumarate and glycopyrronium bromide in their combined MDI. Thus, it was necessary to perform the first precise, valid and reproducible spectrophotometric methods and isocratic, highly sensitive ion pair chromatographic (IPC) method to resolve GLY in the presence of FF in their raw materials and medicinally pressurized MDI in routine quality control analysis laboratories as well as therapeutic drug monitoring labs (Additional file 1).

## Experimental

### Apparatus

Spectrophotometric analysis was carried out on a Shimadzu (Kyoto, Japan) UV-1601 PC, UV–visible double-beam spectrophotometer with matched 1-cm path length quartz cells. The first derivative and the ratio derivative spectra of both drugs were derived in the wavelength range of 190–400 nm using Δλ = 8 nm for the first derivative of spectra and scaling factor = 10.

The chromatographic assay was carried out using TM Series 200 Chromatograph Perkin Elmer instrument supplied with a 30 μL loop of Rheodyne injector valve and a UV detection adjusted to 210 nm.

pH-Meter (Consort NV P-901) was utilized for pH measurements throughout the work. Millipore filter (Sibata) was used for efficient filtration of mobile phase followed by degassing via Merck degasser (solvent L-7612). Mathematical data processing was performed using Total Chrom Workstation made in Massachusetts, USA.

### Materials and reagents

- All the chemicals, solvents were of HPLC grade and efficiently purified deionized water was used throughout the work.
- Formoterol fumarate and internal standard namely dexamethasone (DEX) were obtained from Sigma-Aldrich chemicals (Steinheim, Germany). Glycopyrronium bromide authentic powder was gifted by Novartis (Switzerland). They were analyzed as given without applying further purification steps.
- Bevespi aerosphere^®^; market labeled to eject 4.8 μg FF and 9.0 μg GLY/inhalation (delivered dose), Foradil Breezhaler^®^ contain 4.8 μg of FF/inhaler capsule and Seebri Breezhaler^®^ contain 50 μg of GLY/inhaler capsule were obtained from commercial sources.
- Sodium dodecyl sulphate (SDS), orthophosphoric acid (OPA), methanol and acetonitrile consumed throughout the work were HPLC grade and obtained from Sigma-Aldrich Chemie GmbH chemicals.

### Chromatographic conditions

Knauer C_18_ column (150 mm × 4.6 mm with 5.0 µm particle size) was applied for efficient resolution of FF and GLY at room temperature.

The components of mobile phase are carefully made to acetonitrile (60% v/v): deionized water (40% v/v) and the ion pair SDS percentage of 0.025% added to the mobile phase and the final pH of 3.0 was adjusted using 0.02 M OPA solution. An ultrasonic bath was utilized for mixing the mobile phase run components homogeneously for 30 min, followed by membrane filtration through 0.45 µm filter unit (Ireland), the gases were carefully ejected using an ultrasonic bath for 10 min. The elution rate of mobile phase was adjusted to 1.2 mL/min coupled with reproducible detector readings upon using UV wavelength of 210 nm. The mobile phase was stable within 1 week when kept in refrigerator.

### Standard solution

Ten mg of formoterol fumarate and 20.0 mg of glycopyrronium bromide were added to 100 mL measuring flask containing methanol, to get 100 μg/mL FF stock solution and 200 μg/mL GLY stock solution for both methods. DEX (IS) stock solution (300 μg/mL) was obtained using methanol solvent that was used for further dilution of the stock solutions. Then, either methanol (in the spectrophotometric method) or mobile phase (in the IPC method) was added to achieve the working concentration ranges of combined drug solution. The stock FF and GLY solutions were 4 °C cooled, kept away from light.

### Construction of calibration graphs

For the spectrophotometric methods, the concentration ranges of 0.48–9.6 μg/mL for formoterol fumarate and 0.9–18.0 μg/mL for glycopyrronium bromide were reached by accurate transfer of certain volumes of FF and GLY working solutions into volumetric flasks (10 mL). These flasks were further diluted with methanol to the mark and the zero-order absorption spectra were drawn against methanol.

Using first derivative spectrophotometric analysis, the values were determined at 208.27 nm for FF and 213.27, 239.86 nm for GLY, respectively. Plotting derivative amplitudes against each drug final concentration gave the calibration curve, in turns the equations of regression line could be concluded.

Regarding ratio derivative spectrophotometric analysis, the first derivative of the ratio spectra (the spectra of FF divided by the spectrum of 1.8 µg/mL GLY solution and the spectra of GLY divided by the spectrum of 3.6 µg/mL FF solution) were determined. The amplitudes were measured at 214 or 229 nm for FF and at 240 or 259 nm for GLY, respectively. The calibration curves were obtained upon plotting of derivative amplitudes versus each inhaled drug final concentration, as a result the regression line mathematical equations could be determined.

For the IPC method, transfer specified volumes of FF and GLY working solutions into volumetric flasks (10 mL) to achieve the linearity ranges of 0.048–4.8 and 0.09–9.0 μg/mL of FF and GLY, respectively, then add constant 500 μL DEX stock solution to reach final concentration of 15 μg/mL. Before injection of 30 μL combined drug mixture into HPLC, these flasks were further diluted by the mobile phase and the chromatographic separation temperature was adjusted to 25 °C (ambient temperature). The calibration curves were drawn upon plotting peak area ratio (FF and GLY peak area/DEX peak area) versus the drug concentration, as a result the regression line and mathematical equations could be constructed.

### Analysis of the formoterol fumarate/glycopyrronium bromide synthetic mixtures

For all proposed methods, transfer aliquots of FF and GLY standard solutions preserving the therapeutic ratio of 1:1.875 into 10 mL volumetric flasks series. Then apply the same procedures described under “construction of calibration graphs” followed by calculating % recoveries using the calibration graphs, or the corresponding regression equations.

### Analysis of FF and GLY in their combined metered-dose inhaler

Ten doses of Bevespi aerosphere were delivered via vacuum into 10 mL of methanol for spectrophotometric methods and into 10 mL of mobile phase spiked with 300 μL DEX stock solution for IPC methods to give working solutions containing 0.48 μg/mL of FF and 0.9 μg/mL of GLY. For the IPC method, before injection of combined drug mixture into the HPLC, 0.45 μm sample filters was utilized for accurate filtration of all analyzed samples. Then apply the same procedures described under “Construction of calibration graphs” to analyze three different concentration of FF and GLY carefully selected from their working concentration. Finally, calculate the nominal content of each drug in their inhaled pharmaceuticals from the corresponding equations of regression.

## Results and discussion

### First derivative spectrophotometry (DS)

It was observed that FF possesses UV absorption spectra with two λ_max_ at 217 nm and 252 nm, while GLY possesses UV absorption spectra with two λ_max_ at 210 nm and 222 nm in methanol. Simultaneous determination of FF and GLY solution in methanol using conventional UV spectrophotometry showed interference of the zero order absorption spectra between the two drugs which could be resolved by derivative and ratio derivative spectrophotometry in order to analyze the mixture of the two drugs in their pharmaceuticals.

**Fig. 2:**
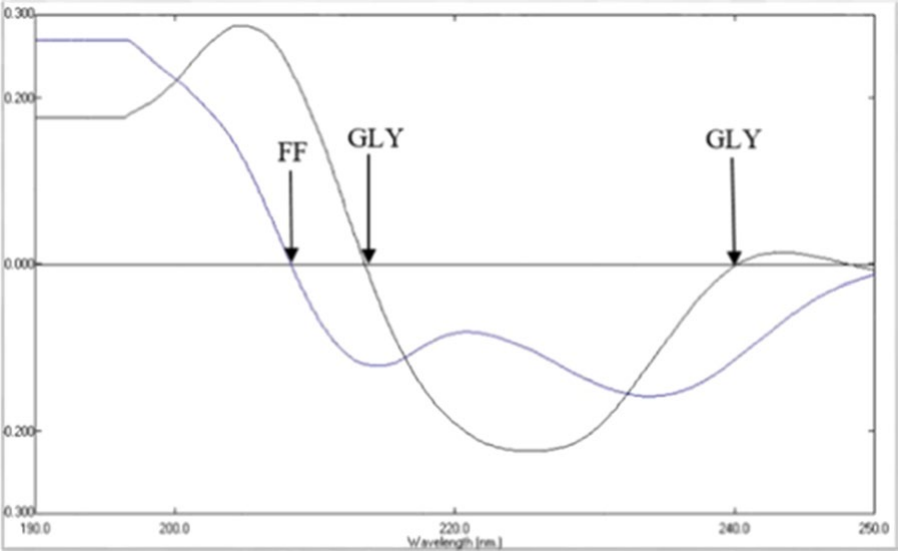
First-order derivative spectra of 7.2 µg/mL FF and 13.5 µg/mL GLY in methanol, Where FF measured at 208.27 nm (at zero crossing for GLY) and GLY measured at 213.27 nm and 239.86 nm (at zero crossing for FF)

Figure 2 displays the first derivative spectra of both drugs by measuring FF first derivative amplitude at the zero crossing of GLY at 208.27 nm (^1^D_208.27_), whereas GLY amplitude was measured at zero crossing point of FF at 213.27 nm and 239.86 nm, respectively (^1^D_213.27, 239.86_) using Δλ = 8 nm and scaling factor = 10.

### Ratio first derivative spectrophotometry (RDS)

**Fig. 3:**
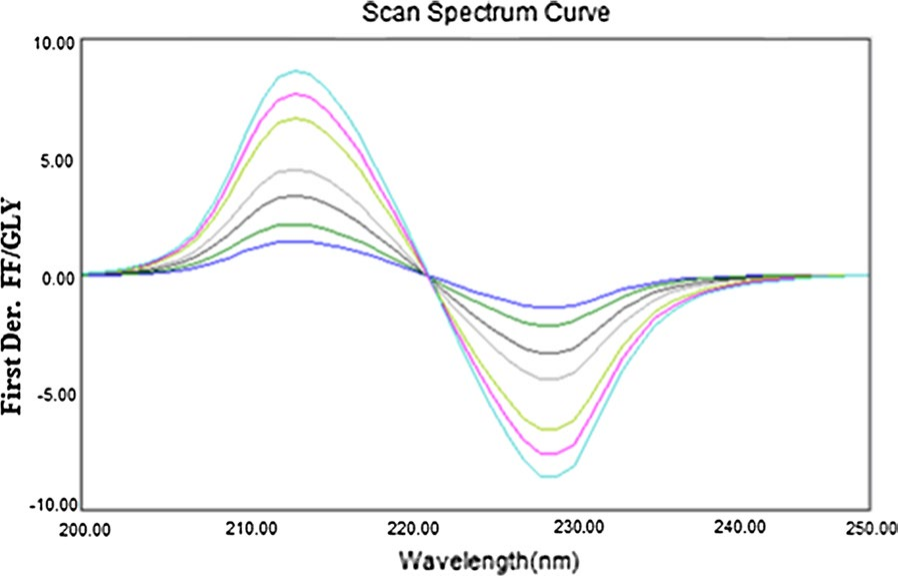
First derivative of the ratio spectra of FF (0.48, 0.96, 1.2, 2.4, 4.8, 7.2 and 9.6 µg/mL) when 1.8 µg/mL GLY was used as divisor (at 214, 229 nm)

Figure 3 displays the first derivative of the ratio spectra of different concentrations of FF standards (FF spectra divided by the spectrum of a solution containing 1.8 µg/mL of GLY). In this figure, the amplitude at 214 or 229 nm (^1^DD_214, 229_) in the ratio derivative spectra corresponds to FF present in the solution.

**Fig. 4:**
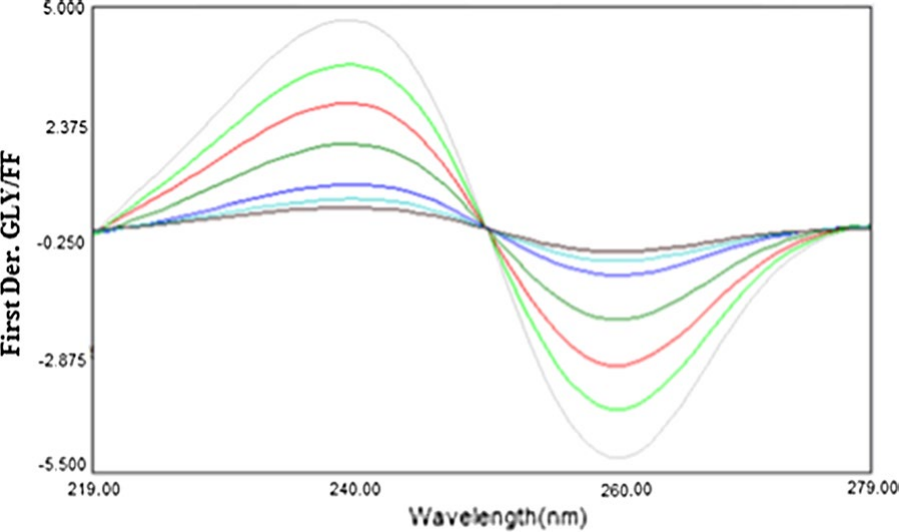
First derivative of the ratio spectra of GLY (0.9, 1.8, 2.25, 4.5, 9, 13.5 and 18.0 µg/mL) when 3.6 µg/mL FF was used as divisor (240, 259 nm)

Likewise, Fig. 4 explains the first derivative of the ratio spectra of different concentrations of GLY standards (GLY spectra divided by the spectrum of 3.6 µg/mL FF solution), on the basis of which, GLY could be quantified by measuring the amplitude at 240 or 259 nm (^1^DD_240, 259_).

The effect of Δλ for the analysis of the first derivative of the ratio spectra was tried to get the ideal wavelength interval; Δλ = 8 nm was selected as appropriate for both drugs. For choosing the standard solution as mathematical divisor, separate concentrations were analyzed and their subsequent calibration curves were recorded. The ideal results depending on signal-to-noise ratio, sensitivity and repeatability parameters were acquired by using divisors of the spectra of 1.8 µg/mL FF and 3.6 µg/mL GLY solutions in the calculation of GLY and FF, respectively.

### IPC method

Ion pair chromatographic technique is broadly applied for efficient separation of both polar and non-ionic studied drug mixtures within the same run time [33]. This technique significantly enhances the symmetry of the resolved drug peaks, decreases the elution time coupled with high reproducibility of the results [34].

Different runs were tried without adding SDS as an ion pairing agent. Applying a mixture of 60% V/V deionized water and 40% V/V acetonitrile resulted in decreasing sensitivity with un-retained peak for FF. While using methanol instead of acetonitrile solvent eluted GLY within 14 min and exhibited a widely broad peak for GLY.

**Fig. 5:**
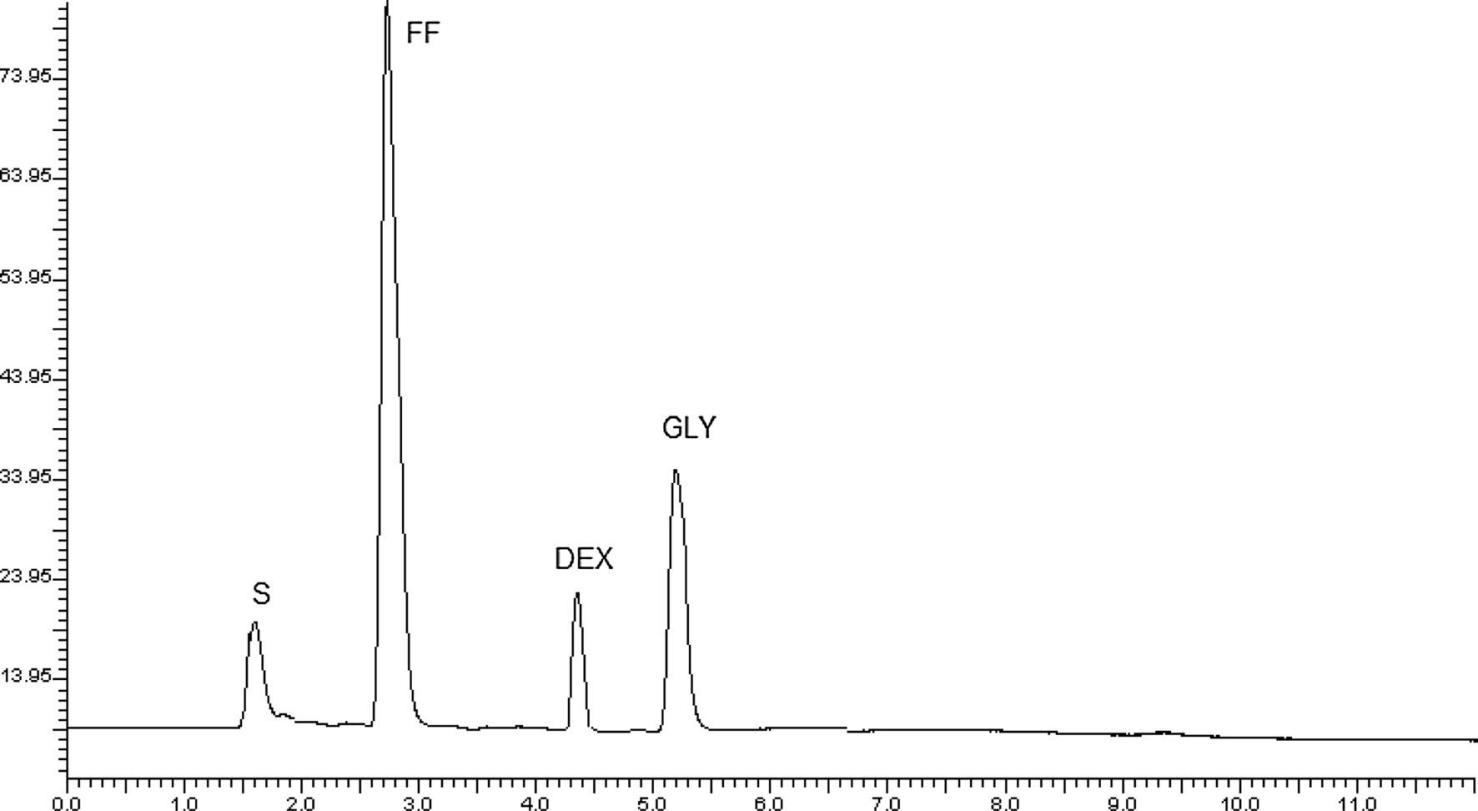
Typical chromatogram for the separation of FF (2.4 µg/mL, 2.7 min), GLY (4.5 µg/mL, 5.2 min.) and DEX (IS; 15 μg/mL, 4.4 min) in synthetic mixture using mobile phase. Chromatographic system: Knauer C_18_ column (150 mm × 4.6 mm with 5.0 µm particle size) Mobile phase mobile phase consisting of acetonitrile: acidified deionized water containing 0.025% SDS pH 3.0 (60: 40% v/v). Flow rate, 1.2 mL/min, UV detection at 210 nm; column temperature, ambient

For the basic studied drugs; FF and GLY, it is critical to select negatively charged ion pairing agents (e.g. alkanesulfonates) to permit efficient separation of the combined drug mixture [33]. SDS is the best ion pairing agent selected due to its availability, economic price and its opposite charge to the basic studied analytes [35]. According to ion pair formation theory, the negatively charged part of SDS interacts with the positively charged analytes [34], while its lipophilic portion interacts with the column stationary phase [34]. As a result, ion pairing agent and the selected FF and GLY form non-charged paired complex that can be reserved and homogenously distributed between different phases consuming run time not exceeds 6 min (Fig. 5).

Critical Micellar concentration (CMC) of SDS at 25 °C is 8.2 mM [35], so the concentration used should be lower than its CMC to avoid the micelle formation (formed in case of using concentration higher than its CMC). IPC is preferred due to its higher separation efficiency accompanied with repeatability of results [34].

**Fig. 6:**
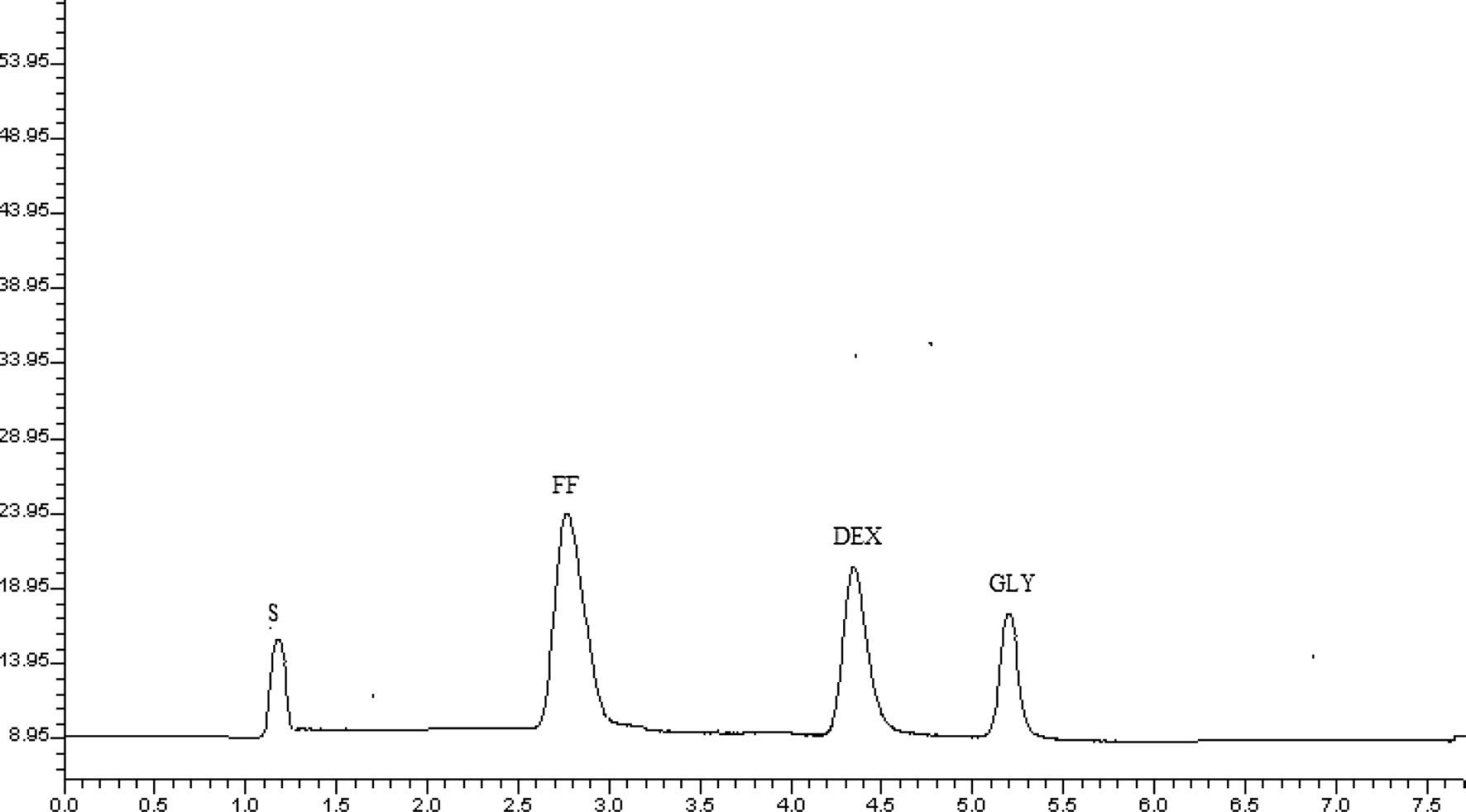
Typical chromatogram for the separation of FF (0.48 µg/mL, 2.7 min), GLY (0.9 µg/mL, 5.2 min) and DEX (IS; 15 μg/mL, 4.4 min) in metered dose inhaler

Figures 5 and 6 exhibited the running chromatogram for FF and GLY solutions enclosing 2.4, 0.48 µg/mL and 4.5, 0.9 µg/mL of each drug in their synthetic mixture and 4.5, 0.9 µg/mL of each drug in synthetic mixture and MDI.

Excellent resolution coupled with high sensitivity was successfully achieved upon adding 0.025% SDS as an effective ion pair reagent to mobile phase. The established IPC elution time not exceeds 6 min; The elution times of FF and GLY were 2.7 and 5.2 min, respectively, as shown in Figs. 5 and 6. This method also enables the quantitative estimation of each drug.

### Method development and optimization

The chromatographic elements that significantly affect the resolution of FF and GLY were examined and considered carefully. Calculation of chromatographic efficiency using; number of theoretical plates, resolution and capacity factor were best described in USP guidelines [36] and the results were shown in Table 1. The pH of the mobile phases was altered over the range 3.0–6.0 using increasing volumes of OPA. The optimum pH of 3.0 was selected for separation and quantitation of both drugs as it provides satisfactory resolution (Rs = 6.55) in short chromatographic run (5.2 min). Various Ratios of acetonitrile: deionized water was investigated. The optimum ratio was found to be (60:40, v/v) acetonitrile: deionized water containing 0.025 SDS at pH 3.0. This system provides the best sensitivity, resolution and the maximum peak efficiency as indicated by N. As given in Table 1, different SDS concentrations within the range (0.015–0.03%) were studied. Using SDS concentration of 0.015% resulted in decreasing the retention time of GLY and un-retained peak for FF. Meanwhile, it was found that increasing SDS concentration, increased the separation efficiency of both drugs as indicated by increased N up to 0.025%; further increase in SDS concentration up to 0.03% resulted in a slight decrease in N. The optimum SDS concentration was 0.025% as it provides the best sensitivity, reasonable run time and peak efficiency and selectivity (Table 1).

**Table 1:** Optimization of the chromatographic parameters for separation of mixture of FF and GLY by the proposed IPC method

Parameter		No. of theoretical plates (N)		Mass distribution ratio (Dm)		Resolution (Rs)	Relative retention (α)
		FF	GLY	FF	GLY	Rs	PP
pH	3.0	3274	2981	1.19	2.96	6.55	2.49
	4.0	1213	2630	1.15	2.82	5.0	2.45
	5.0	1024	2081	1.1	2.66	4.82	2.41
	6.0	887	1673	1.08	2.65	4.39	2.45
Organic modifier type	Acetonitril	3274	2981	1.19	2.96	6.55	2.49
	Methanol	2036	1285	0.89	1.17	5.6	1.31
Ratio of [acidified water: acetonitrile] 0.025% SDS	(30:70)	Un retained peak					
	(40:60)	3247	2981	1.19	2.96	6.55	2.49
	(45:55)	2200	2410	1.27	3.05	4.92	2.39
	(50:50)	2070	725	1.94	4.42	3.75	2.27
	(60:40)	1834	512	2.14	4.78	4.5	2.23
Conc. of SDS %	0.015	Un ret.	1862	0	1.4	0	0
	0.02	1246	2584	0.66	2.0	5.33	3.0
	0.025	3274	2681	1.19	2.96	6.55	2.49
	0.03	1601	1910	0.88	2.61	5.64	2.93

Where: number of theoretical plates (N) = 5.45(t_R_/W_h/2_)^2^

Resolution (R) = 2∆ t_R_/W_1_ + W_2_

Mass distribution ratio (Dm) = t_R _− t_m_/t_m_

Relative retention (α) = Dm_2_/Dm_1_

t_R_ is the retention time of the substance measured from the point of injection and t_M_ is the retention time of a non-retained marker and W_h/2_ is the peak width at the half height

W_1_ and W_2_ are the width of the peaks of the two components at their bases

### Analytical performance

Validation of the established methods were estimated as explained by ICH guidelines [37] (Table 2).

**Table 2:** Analytical performance data for the determination of FF and GLY by the proposed methods

Parameter	FF			GLY			
	DS_208.27nm_	RDS_214/229nm_	IPC_210nm_	DS1_213.27nm_	DS2_239.86nm_	RDS_240/259nm_	IPC_210nm_
Conc. range (μg/mL)	0.48–9.6	0.48–9.6	0.048–4.8	0.9–18	0.9–18	0.9–18	0.09–0.9
r	0.9999	0.9999	0.9999	0.9999	0.9999	0.9999	0.9999
Slope	0.033	0.87	20.8	0.01	0.013	0.36	49.26
Intercept	− 0.014	0.99	− 0.22	0.02	0.002	− 0.23	3.76
LOD (μg/mL)	0.06	0.04	0.018	0.15	0.08	0.17	0.051
LOQ (μg/mL)	0.19	0.1	0.055	0.46	0.28	0.52	0.154
S_*y/x*_	9.00 × 10^−4^	1.4 × 10^−3^	0.19	7.00 × 10^−4^	4.00 × 10^−4^	26.6 × 10^−3^	1.31
S_*a*_	6.00 × 10^−4^	1.00 × 10^−3^	0.11	5.00 × 10^−4^	3.00 × 10^−4^	18.6 × 10^−3^	0.76
S_*b*_	1.00 × 10^−4^	2.00 × 10^−4^	0.04	4.80 × 10^−5^	2.7 × 10^−5^	1.8 × 10^−3^	0.16
% RSD	0.49	0.18	0.63	0.69	0.35	0.63	0.54
% Error	0.15	0.06	0.2	0.22	0.11	0.2	0.17

N.B.—Sy/x = standard deviation of the residuals

Sa = standard deviation of the intercept of regression line

Sb = standard deviation of the slope of regression line

% Error = RSD%/√n

#### Linearity

The calibration curves were constructed by plotting either the derivative, ratio derivative amplitude or the peak area ratio [drug/IS] against each drug concentration in µg/mL for the spectrophotometric or the IPC method, respectively. The graphs were found to be rectilinear over the concentration ranges mentioned in Table 2.

The subsequent equations were assumed by data analysis of the regression line (Table 2).

First derivative spectrophotometry:

**Table:** 

DA = − 0.14 + 0.033 C (r = 0.9999)	For FF at 208.27 nm (^1^D_208.27_)
DA = 0.02 + 0.01 C (r = 0.9999)	For GLY at 213.27 nm (^1^D_213.27_)
DA = 0.002 + 0.013 C (r = 0.9999)	For GLY at 239.86 nm (^1^DD_239.86_)

Ratio first derivative method:

**Table:** 

DA = 0.99 + 0.87 C (r = 0.9999)	For FF at 233 nm (^1^DD_214_)
DA = − 0.23 + 0.36 C (r = 0.9999)	For GLY at 260 nm (^1^DD_240_)

Where DA is derivative amplitude of the spectra at the cited wavelength, C is the concentration of the drug (μg/mL) and r is correlation coefficient.

IPC method:

**Table:** 

PA = − 0.22 + 20.8 C (r = 0.9999)	For FF
PA = 3.76 + 49.26 C (r = 0.9999)	For GLY

PA is the Peak area ratio, C is the drug concentration of (μg/mL) and r is correlation coefficient (Table 2).

### Limit of quantification (LOQ) and limit of detection (LOD)

The written equations were utilized to compute LOQ and LOD as suggested by ICH Q2 (R1) [37] (Table 2).

$${\text{LOQ}}\, = \,10\;{\text{Sa/b}}\;{\text{and}}\;{\text{LOD}}\, = \,3.3\;{\text{Sa/b}}$$

where Sa = intercept of the standard deviation and b = the slope of calibration curve.

### Precision

Triplicate trial assay of FF and GLY in their prepared mixture throughout the same day as recommended in intraday precision and for 3 separate successive days as recommended in inter day precision were established applying three altered concentrations of 0.6, 1.2 and 1.8 µg/mL for FF and 1.125, 2.25 and 3.375 µg/mL for GLY for the spectrophotometric methods and 4.8, 6.0 and 7.2 µg/mL for FF and at 6.75, 9.0 and 11.25 µg/mL for GLY for IPC method to confirm the validity of the studied method. The SD and RSD small values (< 2) exhibited the precision as well as % Er small values (< 1) confirmed the method accuracy (Table 3).

**Table 3:** Precision data of determination of the FF and GLY in pure form using (A) spectrophotometric method and (B) IPC method

(A) Spectrophotometric method						
Derivative spectrophotometric method	FF Conc_208.27nm_ (μg/mL)			GLY Conc_239.6nm_ (μg/mL)		
	4.8	6.0	7.2	6.75	9.0	11.25
Intra-day						
${\bar{\text{X}}}$	100.14	99.80	100.03	100.13	99.21	100.33
± SD	0.29	0.61	0.68	0.29	0.20	0.75
% RSD	0.29	0.61	0.68	0.29	0.20	0.75
% Error	0.17	0.35	0.39	0.17	0.12	0.43
Inter-day						
${\bar{\text{X}}}$	100.12	100.24	99.86	100.14	99.60	100.12
± SD	0.21	0.52	0.95	0.75	0.42	0.55
% RSD	0.21	0.52	0.95	0.74	0.42	0.55
% Error	0.12	0.30	0.55	0.43	0.24	0.32

Ratio derivative spectrophotometric method	FF Conc_214/229nm_ (μg/mL)			GLY Conc_240/259nm_ (μg/mL)		
	4.8	6.0	7.2	6.75	9.0	11.25
Intra-day						
${\bar{\text{X}}}$	99.89	100.07	99.82	100.64	100.74	99.91
± SD	0.58	0.85	0.49	0.36	0.29	0.64
% RSD	0.58	0.85	0.49	0.36	0.29	0.64
% Error	0.33	0.49	0.28	0.21	0.17	0.37
Inter-day						
${\bar{\text{X}}}$	100.53	100.08	100.43	99.82	99.44	100.47
± SD	0.23	0.54	0.75	0.96	0.13	0.76
% RSD	0.23	0.54	0.74	0.96	0.13	0.75
% Error	0.13	0.31	0.43	0.55	0.07	0.44

(B) IPC method						
IPC method	FF Conc. (μg/mL)			GLY Conc. (μg/mL)		
	0.6	1.2	1.8	1.125	2.25	3.375
Intra-day						
${\bar{\text{X}}}$	100.89	99.41	99.45	100.03	99.54	99.24
± SD	0.67	0.41	0.66	0.60	0.66	0.35
% RSD	0.66	0.41	0.66	0.60	0.67	0.35
% Error	0.38	0.24	0.38	0.35	0.39	0.20
Inter-day						
${\bar{\text{X}}}$	99.84	99.60	99.48	99.95	100.58	99.66
± SD	0.44	0.37	0.60	0.53	0.62	0.45
% RSD	0.44	0.37	0.61	0.53	0.61	0.45
% Error	0.23	0.21	0.35	0.30	0.38	0.26

### Accuracy

Student’s t-test and variance ratio *F*-test [38] were utilized to compare the results of assay of FF and GLY in MDI applying derivative spectrophotometry, ratio derivative spectrophotometry plus IPC methods and the comparison chromatographic method for the studied mixture. The outcomes in Tables 4, 5 and 6 showed no significant variance between the mentioned and comparison analytical methods regarding precision and accuracy. The comparison chromatographic method [39] relies on the assay of FF and GLY utilizing C_18_ column and the mobile phase composed of 0.1% OPA: methanol (60:40%, V/V), the pH adjusted to 3.0, at 1.0 mL/min flow rate and coupled with UV detection at 220 nm.

**Table 4:** Assay results for the determination of the FF and GLY in their synthetic mixture and MDI by the proposed DS and reference methods

	a. Proposed DS method (synthetic mixture)				Reference method [39]			
	Conc. taken (μg/mL)		% Found^a^		Conc. taken (μg/mL)		% Found^a^	
	FF	GLY	FF	GLY	FF	GLY	FF	GLY
Data	8.4	15.75	100.08	100.12	10.0	18.0	98.24.	99.58
	7.2	13.5	99.48	100.82	8.4	15.0	99.42	98.35
	6	11.25	99.56	100.40	7.8	12.0	100.13	101.35
	4.8	9	100.21	100.35	6.2	9.0	99.25	100.89
	3.6	6.75	100.03	99.49	4.5	6.0	100.47	99.36
	2.4	4.5	99.99	99.18	2.9	3.0	100.34	100.21
${\bar{\text{X}}}$			99.89	100.06			99.92	99.96
± SD			0.29	0.61			0.55	1.1
t-value			0.32(10.0)	0.20 (10.0)				
*F*-value			3.62 (5.5)	3.16 (5.5)				

	b. Proposed DS method (MDI)				Reference method [39]			
	Conc. taken (μg/mL)		% Found^a^		Conc. taken (μg/mL)		% Found^a^	
	FF	GLY	FF	GLY	FF	GLY	FF	GLY
Data	8.4	15.75	100.89	100.88	10.0	18.0	99.21	100.28
	7.2	13.5	99.52	100.25	8.4	15.0	98.90	99.35
	6	11.25	100.35	100.54	7.8	12.0	100.51	98.52
	4.8	9	99.87	99.35	6.2	9.0	99.54	101.35
	3.6	6.75	100.35	100.48	4.5	6.0	100.37	100.47
	2.4	4.5	100.08	99.36	2.9	3.0	98.50	98.52
${\bar{\text{X}}}$			100.18	100.14			99.51	99.75
± SD			0.47	0.67			0.33	1.14
t-value			1.41 (10.0)	0.74 (10.0)				
*F*-value			3.24 (5.5)	3.17 (5.5)				

Nominal content for GLY in Bevespi^®^ aerosphere by the proposed method = 9.0 μg/buff

Each result is the average of three separate determinations

^a^Nominal content for FF in Bevespi^®^ aerosphere by the proposed method = 4.8 μg/buff

**Table 5:** Assay results for the determination of the FF and GLY in their synthetic mixture by the proposed RDS and reference methods

	Proposed RDS method (synthetic mixture)				Reference method [39]			
	Conc. taken (μg/mL)		% Found^a^		Conc. taken (μg/mL)		% Found^a^	
	FF	GLY	FF	GLY	FF	GLY	FF	GLY
Data	7.8	15.25	99.24	99.41	11.0	19.0	99.42	101.25
	6.6	13.0	100.351	99.97	9.4	16.0	98.24	100.58
	5.4	10.75	99.54	100.89	8.8	13.0	99.31	100.36
	4.2	8.5	99.42	100.87	7.2	10.0	100.45	99.34
	3.0	6.25	100.94	100.39	5.5	7.0	99.78	99.24
	1.8	4.0	99.67	99.77	3.9	4.0	98.54	98.36
${\bar{\text{X}}}$			99.86	100.22			99.29	99.86
± SD			0.65	0.60			0.81	1.6
t-value			1.35 (10.0)	0.73 (10.0)				
*F*-value			1.54 (5.5)	3.08 (5.5)				

	Proposed RDS method (MDI)				Reference method [39]			
	Conc. taken (μg/mL)		% Found^a^		Conc. taken (μg/mL)		% Found^a^	
	FF	GLY	FF	GLY	FF	GLY	FF	GLY
Data	7.8	15.25	99.25	100.87	11.0	7.93	99.02	100.05
	6.6	13.0	100.74	99.68	9.4	7.98	99.21	101.35
	5.4	10.75	100.92	99.35	8.8	8.00	98.21	98.35
	4.2	8.5	100.24	100.97	7.2		99.74	99.65
	3.0	6.25	99.52	100.22	5.5		100.38	99.35
	1.8	4.0	100.34	100.98	3.9		99.17	98.35
${\bar{\text{X}}}$			100.17	100.35			99.29	99.52
± SD			0.66	0.71			0.73	1.13
t-value			2.19 (10.0)	1.52 (10.0)				
*F*-value			1.21 (5.5)	2.55 (5.5)				

Nominal content for GLY in Bevespi^®^ aerosphere by the proposed method = 9.0 μg/buff

Each result is the average of three separate determinations

^a^Nominal content for FF in Bevespi^®^ aerosphere by the proposed method = 4.8 μg/buff

**Table 6:** Assay results for the determination of the FF and GLY in their synthetic mixture by the proposed IPC and reference methods

	Proposed IPC method (synthetic mixture)				Reference method [39]			
	Conc. taken (μg/mL)		% Found^a^		Conc. taken (μg/mL)		% Found^a^	
	FF	GLY	FF	GLY	FF	GLY	FF	GLY
Data	3.6	6.75	99.42	100.12	9.6	13.5	99.38	99.58
	3.0	5.625	99.34	100.82	7.2	11.25	98.34	98.32
	2.4	4.5	100.35	100.4	4.8	9.0	99.25	99.25
	1.8	3.375	100.97	100.35	3.6	6.75	100.38	99.87
	1.2	2.25	99.57	99.49	2.4	4.5	99.07	100.77
	0.6	1.125	99.34	99.18	1.2	2.25	98.34	100.82
${\bar{\text{X}}}$			99.83	100.06			99.13	99.77
± SD			0.68	0.61			0.76	0.95
t-value			1.7 (10.0)	0.63 (10.0)				
*F*-value			1.27 (5.5)	2.41 (5.5)				

	Proposed IPC method (MDI)				Reference method [39]			
	Conc. taken (μg/mL)		% Found^a^		Conc. taken (μg/mL)		% Found^a^	
	FF	GLY	FF	GLY	FF	GLY	FF	GLY
Data	3.6	6.75	99.87	100.52	9.6	13.5	99.94	100.85
	2.7	5.625	99.68	100.33	7.2	11.25	99.88	100.35
	1.8	4.5	99.35	100.08	4.8	9.0	100.94	100.39
	0.9	3.375	100.87	99.52	3.6	6.75	99.72	98.33
	0.45	2.25	100.24	99.68	2.4	4.5	99.40	99.58
	0.36	1.125	99.34	100.48	1.2	2.25	99.24	99.07
${\bar{\text{X}}}$			99.89	100.1			99.85	99.76
± SD			0.59	0.42			0.60	0.95
t-value			0.11 (10.0)	0.8 [10]				
*F*-value			1.04 (5.5)	5.06 (5.5)				

Nominal content for GLY in Bevespi^®^ aerosphere by the proposed method = 9.0 μg/buff

Each result is the average of three separate determinations

^a^Nominal content for FF in Bevespi^®^ aerosphere by the proposed method = 4.8 μg/buff

### Robustness

The consistence of the peak area measured values with the minor intended changes in the testing parameters was evaluated by the established IPC method. These parameters include; pH (3.0 ± 0.1), acetonitrile: acidified water mobile phase proportional ratio (60:40 ± 2% v/v), and SDS added concentration (0.025 ± 0.0025% w/v). These minor changes not negatively affect the peak area of both drugs.

### Selectivity and specificity

Specificity of the proposed methods were assessed by the standard addition method on the dosage form. Known amounts of FF and GLY standards have been added at different concentrations (FF: 1.2, 2.4, 4.8 μg/mL; GLY: 2.25, 4.5, 9.0 μg/mL). Triplicate assay was performed at each concentration level. Measurement of the percent recovery of the added analytes amount to the sample gave accuracy as given in Table 7 prove the accuracy of the proposed methods within the desired range.

**Table 7:** Accuracy of the proposed methods using standard addition method

Added concentration to Bevespi^®^ aerosphere^®^ 0.48, 0.9 (μg/mL)		% Recovery^a^					
		FF			GLY		
FF	GLY	DS	RDS	IPC	DS	RDS	IPC
1.2	2.25	100.51	99.1	100.95	99.1	99.47	100.26
2.4	4.5	99.83	99.83	100.32	99.79	100.55	99.75
4.8	9.0	99.31	100.43	99.24	100.3	99.84	100.63
Mean ± RSD %		99.88 ± 0.49	99.81 ± 0.55	100.1 ± 0.72	99.76 ± 0.50	99.93 ± 0.45	100.13 ± 0.4

Each result is the average of three separate determinations

^a^Nominal content for FF Bevespi^®^ aerosphere by the proposed method = 9.0 μg/buff, Nominal content for GLY in Bevespi^®^ aerosphere by the proposed method = 4.8 μg/buff, assay was performed on three levels, each result is the average of three separate determinations

### Analysis of formoterol fumarate/glycopyrronium bromide in synthetic mixtures and co-formulated metered dose inhaler

Analysis of FF and GLY in pressurized MDI in their recommended therapeutic doses of 4.8/9, respectively was performed applying the proposed methods in their pressurized metered dose inhaler and synthetic mixtures. The results found in Tables 4, 5 and 6 are reproducible and accurate as given by the good % recoveries which enable precise analysis of FF and GLY in the quality control labs. The high specificity of the proposed method was revealed as the inhaler propellant, hydrofluoroalkane that was repelled throughout inhalation has negligible effect on the inhaled drugs measured in MDI.

## Conclusion

Simple and valid spectrophotometric and liquid chromatographic methods were carried out successfully for the analysis of formoterol fumarate and glycopyrronium bromide in their raw materials as well as pharmaceutical metered dose inhaler. The developed spectrophotometric methods for the simultaneous analysis of FF and GLY are simple, available, and time saving. Highly sensitive and accurate ion-pairing liquid chromatographic method was successfully established for simultaneous estimation of both FF and GLY in pure form as well as in the MDI with short run time not exceeds 6 min and good resolution (R_s_ > 2). Compared with the studied spectrophotometric method, the IPC method is highly sensitive and specific. The % RSD and LOD are satisfactorily good for quality control drug analysis without interference from the common excipients and the ejected propellant of MDI.

## Additional file

**Additional file 1.** Illustrative data and figures that described in detail the optimization of the chromatographic performance and system suitability for HPLC method according to USP guidelines.

## Data Availability

All relevant supporting data are fully available without any restriction.

## Abbreviations

FF: formoterol fumarate
GLY: glycopyrronium bromide
IPC: ion pair chromatography
OPA: orthophosphoric acid
