# Supplementary material for: Multiple analytical methods for determination of formoterol and glycopyrronium simultaneously in their novel combined metered dose inhaler
Source: BMC Chem. 2019 Jun 10;13(1):75. doi: 10.1186/s13065-019-0592-9 (PMC6661743; doi:10.1186/s13065-019-0592-9)
Supplement: Supplementary file 1 — Additional file 1. Illustrative data and figures that described in detail the optimization of the chromatographic performance and system suitability for HPLC method according to USP guidelines. [file 13065_2019_592_MOESM1_ESM.docx]

**Additional Data**

**Optimization of the Chromatographic Performance and System Suitability:**

Most appropriate chromatographic parameters influencing FF and GLY were checked and assayed carefully. USP guidelines [36] gave terms of number of theoretical plates, capacity factor, resolution, relative retention and mass distribution ratio to estimate chromatographic performance as illustrated in table 1.

**Stationary phase**

Two columns were tested for performance investigations, including knaur C_18_ column (150 mm × 4.6 mm, 5 µm particle size), and Onyx monolithic HD column (100 mm × 4.6 mm. 3μm). The investigational study revealed that, symmetrical peaks with an efficient resolution were obtained upon using knaur C_18_ column. Besides, Onyx monolithic HD column was inappropriate to provide well separated peaks in a rational time.

**UV-Detection:**

Detection and resolving the peaks of the studied drugs were investigated using different wavelengths 210, 220 and 230 and 242 nm. The most suitable wavelength for UV detection response was found to be 210 nm due to high sensitivity, particularly for FF permitting estimation of both drugs simultaneously in their co-formulated inhaler without any interference (Figure S1).

**
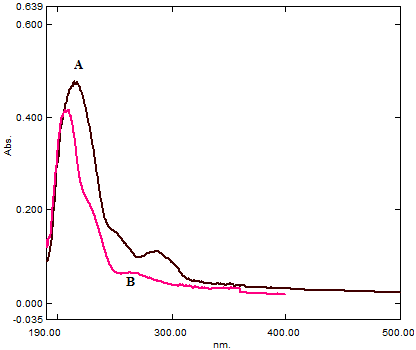
**

**Figure S1.** Absorption spectrum of 4.8 μg/mL FF (A) and 9.0 μg/mL GLY (B) in methanol**.**

**Mobile Phase:**

Chromatographic system performance was studied using many modifications in the composition of the mobile phase. These changes included; pH change, ratio and composition of the mobile phase and the flow rate. The choice of the mobile phase was relied on resolution, peak shape and run time. The results given are shown in table 1.

***pH of the mobile phase:***

The pH of the mobile phases was altered over the range 3.0 to 6.0 using increasing volumes of OPA. Difference in the hydrophobicity and dissociation constants of the two drugs was expressed by their log *P* (octanol/water) and p*Ka* values, respectively. FF has log *P* value of 1.91 and two p*Ka* values of 8.61 and 9.81, while GLY has log *P* value of -1,2 and two p*Ka* value of 11.5 and -4.3 [51]. Negligible effect on the run times of FF and GLY was observed upon increasing the pH value to pH 6.0 as FF and GLY will be completely ionized over the investigated pH range. The increase in the pH from 4.0 to 7.0, resulted in loss of the peak sharpness and peak symmetry. As presented in Table 1, the optimum pH of 3.0 was selected for separation and quantitation of both drugs as it provides satisfactory resolution (Rs = 6.55) in short chromatographic run (5.2 min.) as well as maximum efficiency of the separated peaks as indicated by highest the number of theoretical plates (N) (Figure S2).


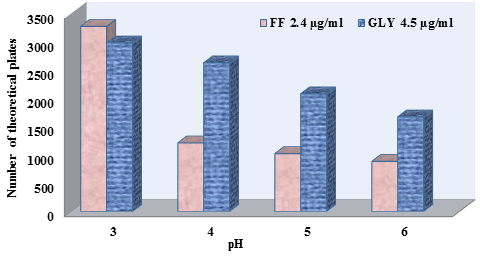


**Figure S2:** Effect of different pH on the number of theoretical plates of FF 2.4 µg/mL and GLY 4.5 µg/mL using mobile phase consisting of acetonitrile: deionized water containing 0.025% SDS (60: 40% v/v). Flow rate, 1.2 mL/min.

***Type of Mobile phase type:***

Different runs were tried without adding SDS as an ion pairing agent. Applying a mixture of 60% V/V deionized water and 40% V/V acetonitrile resulted in decreasing sensitivity with un-retained peak for FF (Figure S3). While using methanol instead of acetonitrile solvent eluted GLY within 14 minutes and exhibited a widely broad peak for FF (Figure S4).

**
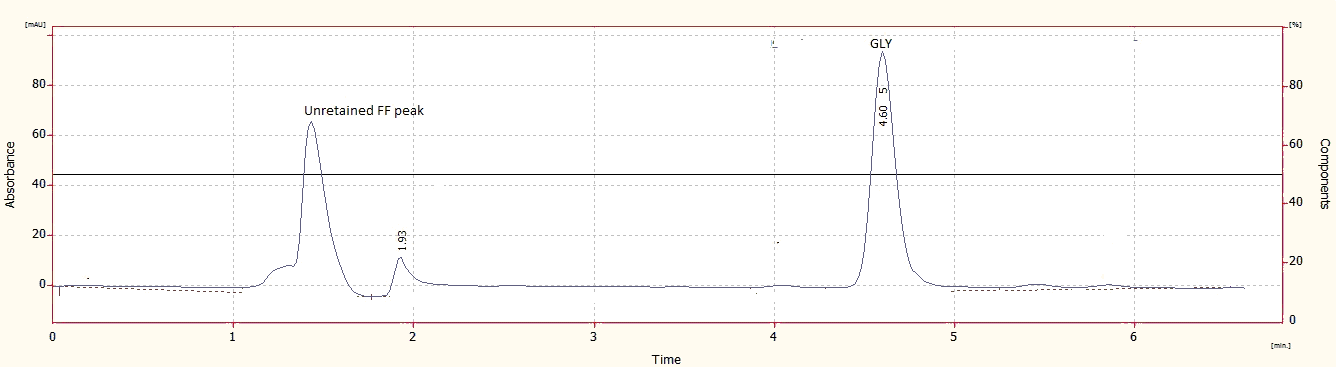
**

**Figure S3:** Chromatogram where acetonitrile was used as mobile phase without SDS.

**
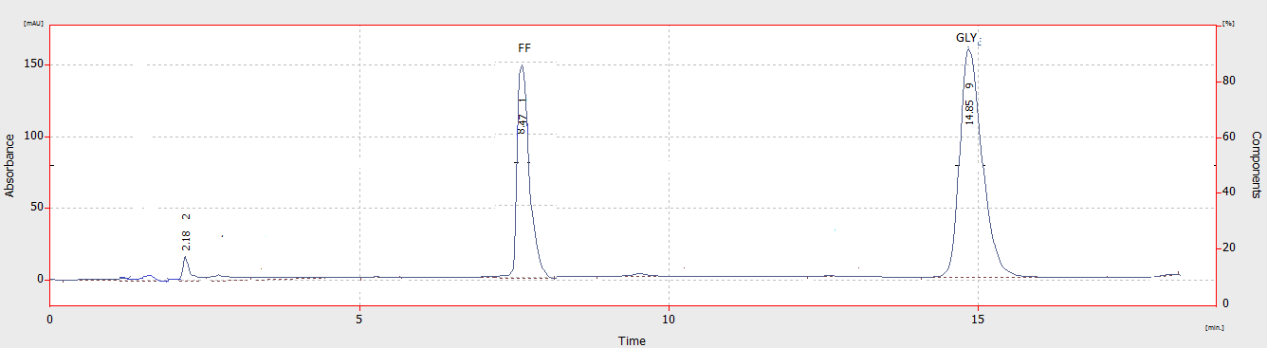
**

**Figure S4:** Chromatogram where methanol was used as mobile phase without SDS.

***Mobile phase type, composition and ratio:***

Various Ratios of acetonitrile: deionized water was investigated. Using the ratio of acetonitrile to water (70:30, v/v), the retention time of the two drugs was sharply decreased causes un-retained FF peak. While using the ratio of acetonitrile to water (40:60, v/v), the retention time of the two drugs was highly increased with a decrease in the separation efficiency as indicated by a sharp reduction in N (Table 1). The optimum ratio was found to be (60:40, v/v) acetonitrile: deionized water containing 0.025 SDS at pH 3.0. This system provides the best sensitivity, resolution and the maximum peak efficiency as indicated by N (Figure S5).

**
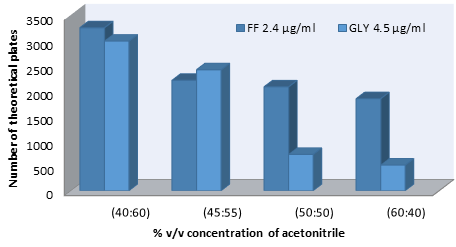
**

**Figure S5.** Effect of different % v/v concentration of acetonitrile on the number of theoretical plates of FF 2.4 µg/mL and GLY 4.5 µg/mL using mobile phase containing 0.025% SDS at pH 3.0.and Flow rate, 1.2 mL/min.

***Concentration of SDS***

As given in Table 1, different SDS concentrations within the range (0.015 % -0.03 %) were studied. Using SDS concentration of 0.015 % resulted in decreasing the retention time of GLY and un-retained peak for FF. Meanwhile**,** it was found that increasing SDS concentration, increased the separation efficiency of both drugs as indicated by increased N up to 0.025 %; further increase in SDS concentration up to 0.03 % resulted in a slight decrease in N (Figure S6). The optimum SDS concentration was 0.025 % as it provides the best sensitivity, reasonable run time and peak efficiency and selectivity.


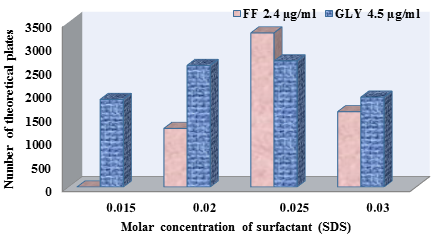


**Figure S6.** Effect of different molar concentration of surfactant (SDS) on the number of theoretical plates of FF 20.0 µg/mL and GLY 10.0 µg/mL using mobile phase consisting of acetonitrile: deionized water pH 3.0 (60: 40% v/v). Flow rate, 2.0 mL/min.

***Flow rate:***

The flow rate was altered within range of 0.8 – 1.4 mL/min to study its effect on the peak separation of the mentioned drugs. A flow rate of 1.2 mL/min was ideal to separate both drugs within 6 min. Also, at high flow rates (1.4 mL/min), short run time was gained but accompanied with a marked decrease in sensitivity. In contrast, lower flow rate (0.6 mL/min) was not accepted as it leads to marked increase in the retention time.

***Internal standard selection***

The use of I.S. is very important to provide a well-developed precise and accurate analytical method, adjusting injection volume variation and removal of the error produced by adsorption on the stationary phase or by interference of the sample matrix. For selecting a suitable internal standard, several drugs were tried such as dexamethasone (DEX), budesonide, cyproheptadine and levofloxacin. Dexamethasone of concentration of 15 µg/mL was selected as the best IS, giving symmetrical and well separated peaks. The optimum chromatographic conditions for separation of FF and GLY are summarized in table 1.
